# A Strategic Research and Innovation Agenda for personalized prevention: towards a European implementation Roadmap

**DOI:** 10.1093/eurpub/ckag111

**Published:** 2026-07-06

**Authors:** Stefania Boccia, Roberta Pastorino, the PROPHET* Consortium†, Stefania Boccia, Sara Farina, Mario Masiello, Tommaso Osti, Angelo Maria Pezzullo, Angelica Valz Gris, Roberta Pastorino, Patricia Cervera de la Cruz, Mahsa Shabani, Pascal Borry, Eva Van Steijvoort, Claudia Louati, Yasemin Zeisl, Andres Metspalu, Anu Reigo, Maris Teder-Laving, Pragathy Kannan, Helena Kääriäinen, Markus Perola, Charlotte Alcouffe, Eva Fadil, Valeria Fava, Daniela Quaggia, Maria Luis Cardoso, Alexandra Costa, Cristina Costa, Astrid Moura Vicente, Cristina Barahona-López, Beatriz Perez-Gomez, Elena Plans Beriso, Jhon Alvarez Ahlgren, Evelina Flodkvist, Alexandra Gyllenberg, Ingrid Kockum, Stefan Swartling Peterson, Carl Johan Sundberg, Laura Blackburn, Chantal Babb de Villiers, Magda Chegkazi, Arshiya Merchant, Serena Scollen, Julian Hein, Martina Cornel, Loes Lindiwe Kreeftenberg, Carla van El, Manuela Pausan, Melanie Goisauf

**Affiliations:** Section of Hygiene, Department of Life Sciences and Public Health, Università Cattolica del Sacro Cuore, Rome, Italy; Department of Woman and Child Health, Fondazione Policlinico Universitario A. Gemelli IRCCS, Rome, Italy; Section of Hygiene, Department of Life Sciences and Public Health, Università Cattolica del Sacro Cuore, Rome, Italy; Department of Woman and Child Health, Fondazione Policlinico Universitario A. Gemelli IRCCS, Rome, Italy

The rapid evolution of omics technologies, combined with the integration of digital health resources, has ushered in a new era of medicine. This ‘precision revolution’ has created unprecedented opportunities for personalized prevention by enabling the identification of individual risk profiles through the integration of multiple sources of health-related data and information into preventive decision-making. This is particularly relevant for chronic diseases, whose multifactorial nature and major burden on healthcare systems may benefit from more accurate risk stratification integrated into population-based prevention strategies [1].

Personalized prevention aims to refine and personalize existing preventive strategies, including cancer screening programs, preventive pharmacological interventions and lifestyle-based prevention, by moving beyond traditional risk stratification approaches based primarily on factors such as age and sex. By integrating additional variables, including omics information, environmental exposures, socio-economic and behavioural determinants, personalized prevention has the potential to better tailor preventive interventions to individual risk profiles. Yet, while its potential to improve population health and reduce healthcare burden is widely recognized, its implementation raises unresolved questions regarding effectiveness, cost-effectiveness, equity, and governance. The main challenge is therefore no longer the advancement of technologies themselves, but their effective integration into healthcare systems and routine clinical and public health practice, which currently remain fragmented and inconsistent across Europe [2].

Importantly, even technologies supported by robust evidence and incorporated into international clinical guidelines remain unevenly implemented across Europe. Examples in the field of omic-based preventive strategies include cascade testing for hereditary cancers, such as *BRCA1/2* testing for hereditary breast and ovarian cancer prevention, and pharmacogenetic testing for *DPYD* variants to guide fluoropyrimidine therapy in colorectal cancer treatment. Although these applications are supported by major scientific societies and represent some of the most clinically validated examples of personalized cancer prevention, their integration into national and regional public health strategies remains heterogeneous across Europe [3]. The same applies to non-omics personalized prevention approaches, including risk-stratified screening strategies based on lifestyle-related exposures, such as annual low-dose computed tomography screening for individuals with a significant smoking history, despite the growing evidence supporting its effectiveness in reducing lung cancer mortality [4].

In contrast, emerging approaches that leverage on omic-based technologies such as polygenic risk scores still lack sufficient evidence regarding clinical utility, cost-effectiveness, and population-level impact, further complicating their implementation into routine prevention strategies. Together, these examples suggest that both the availability of robust evidence and the existence of clinical utility are insufficient, on their own, to ensure large-scale implementation [3]. Organizational capacity, governance frameworks, interoperable infrastructures, reimbursement policies, and workforce preparedness remain equally critical determinants of adoption. Although risk stratification may improve the targeting of preventive interventions, it may also exacerbate health inequalities if access to testing, follow-up care and preventive services is not equitably ensured. At the same time, expanding risk stratification may increase overdiagnosis, surveillance burden and medicalization among otherwise healthy individuals if implementation is not supported by robust evidence and proportionate benefit. Moreover, translating complex health and omics information derived from large-scale datasets into actionable prevention strategies requires validated pathways for interpretation, communication, and implementation, as well as robust governance frameworks capable of ensuring data privacy and maintaining public trust [5]. These tensions highlight that personalized prevention is not solely a scientific and technological endeavour, but also a societal and policy challenge.

In this context, the PROPHET (PeRsOnalized Prevention roadmap for the future HEalThcare) project developed a Strategic Research and Innovation Agenda (SRIA) and an accompanying Roadmap aimed at identifying the main barriers, priorities and implementation needs for integrating personalized prevention into European health systems [2, 6]. In order to stimulate reflection on the structural conditions required for translating personalized prevention into routine public health practice, during the development of the PROPHET consortium we focused primarily on genomics and other omics-based technologies, in line with its positioning within the International Consortium for Personalised Medicine (ICPerMed) [7]. Based on literature mapping [3], consortium input and stakeholder engagement [8, 9], the SRIA identified ten challenges for the implementation of personalized prevention [10]. A summary of these challenges, together with the main gaps and strategic objectives, is presented in Table 1. An urgent priority is to strengthen investment in evidence-based prevention, which, despite its well-recognized long-term benefits in improving population health and reducing healthcare costs, continues to receive insufficient attention and funding across many European health systems. In a context of constrained public budgets and competing health priorities, scaling up personalized prevention will inevitably require prioritization choices and careful evaluation of its added value compared with existing public health interventions. This will require robust evidence on population-level effectiveness, cost-effectiveness, and long-term sustainability to ensure that personalized approaches contribute meaningfully to broader public health impact rather than remaining confined to niche applications. Beyond investments, advancing personalized prevention will also require interoperable and secure data infrastructures and stronger evidence synthesis mechanisms. In particular, the development of interoperable and secure data ecosystems should be aligned with broader European initiatives such as the European Health Data Space (EHDS) and the Genomic Data Infrastructure (GDI), which may support cross-border data sharing, harmonization, and integration of health and omics data across European healthcare systems.

**Table 1.** A summary overview of the 10 challenges of the PROPHET SRIA, key gaps for personalized prevention implementation and strategic objectives to overcome.

Challenge	Key gaps	Strategic objectives
1. Scope of health promotion and prevention	Health systems remain predominantly treatment-oriented, with fragmented prevention policies and limited cross-sector coordination	Strengthen governance of health determinants across sectors and increase investments in preventive policies and integrated pilot initiatives
2. Continuous evidence synthesis for personalized prevention	Limited evidence on the effectiveness and cost-effectiveness of personalized prevention strategies. Lack of systematic evidence synthesis mechanisms	Develop continuous evidence synthesis systems and promote research generating robust evidence on clinical utility and public health impact
3. Implementation of the PROPHET framework	Absence of shared frameworks to guide evaluation and implementation of personalized prevention strategies	Promote adoption of the PROPHET framework and establish coordinated European networks supporting evidence-based implementation
4. Data collection, integration and infrastructure	Fragmented and non-interoperable health and omics data systems, with barriers to cross-border data sharing	Develop interoperable data infrastructures and harmonized standards to support integration and secure sharing of health and omics data
5. Community engagement and trust	Limited public awareness, health literacy and digital literacy affecting participation in personalized prevention initiatives	Promote communication strategies, citizen engagement and initiatives aimed at strengthening trust in personalized prevention
6. Involvement of health professionals and policy makers	Insufficient training and limited integration of scientific evidence into policy-making processes	Strengthen professional education and promote collaboration between researchers, clinicians and decision-makers
7. Regulatory frameworks and collaboration with the private sector	Fragmented regulatory landscape and limited structured public–private collaboration	Develop transparent governance models for public–private partnerships supporting personalized prevention
8. Access, equity and coverage	Inequalities in access to personalized prevention services across regions and population groups	Promote equitable access to technologies and services through inclusive and territorially balanced healthcare models
9. Ethical, legal and social issues (ELSI)	Variability in ethical and legal governance of health and genomic data across countries	Strengthen harmonized ethical frameworks and ensure appropriate safeguards for data protection and non-discrimination
10. Behaviour change	Risk communication alone is insufficient to promote sustained behavioural change	Develop personalized behavioural interventions and tools supporting long-term lifestyle changes

Further actions should address the limited preparedness of both healthcare professionals and citizens to engage with increasingly complex personalized prevention approaches. European health systems will need to strengthen specialized professional competencies, including expertise in genetic counselling and risk communication, while also promoting greater public awareness and participation in prevention programmes and population-based research initiatives. Improving genomic and digital health literacy through targeted education and communication strategies will be essential to support informed decision-making, foster public trust and ensure equitable implementation across different population groups. At the same time, persistent inequalities in access, together with unresolved ethical, legal and regulatory issues related to health and genomic data, further complicate implementation. Additional concerns relate to the integration of personalized prevention within broader health promotion strategies and to the need for transparent and balanced forms of collaboration with the private sector.

Importantly, addressing these barriers will require more than coordinated research efforts. The feasibility of implementation will largely depend on political commitment, resource allocation and the ability of health systems to integrate innovation into already resource-constrained prevention frameworks. In this respect, the accompanying PROPHET Roadmap should be considered a call to action for national Ministries of Health, European institutions, funding programs and public health stakeholders to promote a coordinated and sustainable integration of personalized prevention across Europe.

In conclusion, personalized prevention represents a promising but still underdeveloped component of European public health. Strategic agendas such as the PROPHET SRIA and Roadmap may provide a useful starting point to orient future research and policy discussions. However, their ultimate impact will depend on the capacity of European health systems to translate these approaches into policies and practices that are effective, equitable, and sustainable.

## Supplementary Material

ckag111_Supplementary_Data

## Data Availability

No new data were generated or analysed in support of this Commentary. The manuscript is based on previously published literature and publicly available project outputs.
