# Supplementary material for: A Strategic Research and Innovation Agenda for personalized prevention: towards a European implementation Roadmap
Source: Eur J Public Health. 2026 Jul 6;36(4):ckag111. doi: 10.1093/eurpub/ckag111 (PMC13335471; doi:10.1093/eurpub/ckag111)
Supplement: ckag111_Supplementary_Data [file ckag111_supplementary_data.docx]

***Members of PROPHET Consortium:**

Sara Farina (1), Mario Masiello (1), Tommaso Osti (1), Angelo Maria Pezzullo (1), Angelica Valz Gris (1), Martina Cornel (2), Loes Lindiwe Kreeftenberg (2), Carla van El (2), Manuela Pausan (3), Melanie Goisauf (3), Cristina Barahona-López (4), Beatriz Perez-Gomez (4), Elena Plans Beriso (4), Valeria Fava (5), Daniela Quaggia (5), Magda Chegkazi (6), Arshiya Merchant (6), Serena Scollen (6), Andres Metspalu (7), Anu Reigo (7), Maris Teder-Laving (7), Claudia Louati (8), Yasemin Zeisl (8), Julian Hein (9), Pragathy Kannan (10), Helena Kääriäinen (10), Markus Perola (10), Charlotte Alcouffe (11), Eva Fadil (11), Patricia Cervera de la Cruz (12), Mahsa Shabani (12), Maria Luis Cardoso (13), Alexandra Costa (13), Cristina Costa (13), Astrid Moura Vicente (13), Jhon Alvarez Ahlgren (14), Evelina Flodkvist (14), Alexandra Gyllenberg (14), Ingrid Kockum (14), Stefan Swartling Peterson (14), Carl Johan Sundberg (14), Pascal Borry (15), Eva Van Steijvoort (15), Laura Blackburn (16), Chantal Babb de Villiers (16).

**Affiliations**

1. *Università Cattolica del Sacro Cuore (UCSC), Rome, Italy*
2. *Amsterdam University Medical Center (Amsterdam UMC), Amsterdam, the Netherlands*
3. *BBMRI-ERIC, Graz, Austria*
4. *Centro de Investigación Biomédica en Red (CIBER) / Instituto de Salud Carlos III (ISCIII), Madrid, Spain*
5. *Cittadinanzattiva / Active Citizenship Network (ACN), Rome, Italy*
6. *ELIXIR, Heidelberg, Germany*
7. *Estonian Biobank, Institute of Genomics of the University of Tartu (TARTU), Tartu, Estonia*
8. *European Patients Forum (EPF), Brussels, Belgium*
9. *European Public Health Association (EUPHA), Utrecht, the Netherlands*
10. *Finnish Institute for Health and Welfare (THL), Helsinki, Finland*
11. *GAC Group (GAC), Nice, France*
12. *Ghent University (UGENT), Ghent, Belgium*
13. *Instituto Nacional de Saúde Doutor Ricardo Jorge (INSA), Lisbon, Portugal*
14. *Karolinska Institutet (KI), Stockholm, Sweden*
15. *KU Leuven (KUL), Leuven, Belgium*
16. *PHG Foundation (PHGF), Cambridge, United Kingdom*
